# Transcriptomic signature can distinguish chronic neutrophilic leukemia from ambiguous neutrophilic leukemias

**DOI:** 10.3389/fgene.2025.1556519

**Published:** 2025-04-04

**Authors:** Chao Guo, Zhen-Ling Li

**Affiliations:** Department of Hematology, China-Japan Friendship Hospital, Beijing, China

**Keywords:** chronic neutrophilic leukemia, WGCNA, neutrophil, expression, LASSO

## Abstract

**Background:**

Identifying uncommon neutrophilic leukemias presents a challenging task, owing to the analogous morphological characteristics and the dearth of molecular markers. The transcriptomic profile of bone marrow cells in this disease subset has been rarely explored.

**Material and Methods:**

The OHSU-CNL dataset, encompassing clinical parameters and parallel transcriptomic matrix, was downloaded from the Genomic Data Commons (GDC) database. Distinctive co-expressed gene modules and pivotal genes for chronic neutrophilic leukemia (CNL) were identified using R software. Subsequently, a diagnostic model for CNL denoted as CNL-5 was formulated employing least absolute shrinkage and selection operator (LASSO) regression analysis. The diagnostic power of the CNL-5 model was compared with conventional clinical/genetic markers via multi-ROC analysis. The divergence in overall survival between CNL-5 risk groups was delineated by Kaplan–Meier analysis, and the predictive power (AUC and Harrison’s C index) was determined by time-dependent ROC. Cell signaling pathways associated with CNL-5 risk were identified by genomic set enrichment analysis (GSEA).

**Results:**

Neither clinical indicators nor genetic markers were sufficient to classify neutrophilic leukemias. Through weighted gene co-expression network analysis (WGCNA), the brown module was discerned to be CNL-specific (p = 8e−16, R^2^ = 0.5). Using LASSO analysis, the CNL-5 model, with risk scores based on the weighted expression value of five genes (PDCD7/CR2/ZSCAN20/TRIM68/LILRA6) dichotomized patients into CNL-like and Atypical-CNL groups. Compared to the Atypical-CNL group, the CNL-like group demonstrated a clinical phenotype more consistent with CNL and had a significantly higher prevalence of CSF3R mutations (p < 0.05). Additionally, the AUC of the CNL-5 risk model surpassed that of conventional clinical/genetic markers, as validated by the GSE42731 dataset. Poorer survival was revealed in the high-risk group than in the low-risk group defined by the CNL-5 model. GSEA identified CNL-5-associated pathways, such as the inhibition of oxidative phosphorylation and the activation of IL6-JAK-STAT3 signaling.

**Conclusion:**

A novel expression signature-based diagnostic assessment for CNL was developed, which showed better diagnostic utility than conventional indicators.

## Background

A group of rare myeloid neoplasms, such as chronic neutrophilic leukemia (CNL), atypical chronic myeloid leukemia (aCML), chronic myelomonocytic leukemia (CMML), myelodysplastic/myeloproliferative neoplasms, unclassified (MDS/MPN-U), and myeloproliferative neoplasms, unclassified (MPN-U), is characterized by neutrophilia, with or without dysplasia, and exhibits overlapping morphological and genetic features (Zhang et al., 2019; Maxson and Tyner, 2017; Deininger et al., 2017; Arber et al., 2016). CNL is an uncommon myeloid neoplasm, hallmarked by an overabundance of mature neutrophils in both peripheral blood and bone marrow, with approximately 150 cases meeting the current criteria by 2015 (Bain and Ahmad, 2015). Meanwhile, aCML is identified by an elevation in dysplastic neutrophils and their precursors, an appearance mirroring classic BCR-ABL-positive CML, albeit with a significantly lower prevalence (Orazi and Germing, 2008). CMML, another member of the MDS/MPNs, is notable for an increase in blasts and promonocytes. However, in clinical scenarios, some patients with neutrophilia exhibit both myelodysplastic and myeloproliferative features, making diagnosis difficult (Maxson and Tyner, 2017). The molecular markers of CNL, such as the CSF3R T618I mutation, lack specificity and can also be observed in other myeloid neoplasms, such as aCML (Dao et al., 2020) and CMML (Bezerra et al., 2021).

Alongside diagnosis, the treatment and prognosis of these neutrophilic leukemias pose additional challenges. To date, there is no established treatment for CNL. Traditional treatments, for instance, hydroxyurea and interferon-α (IFNα), provide unsatisfactory response durations (Elliott et al., 2005; Bohm and Schaefer, 2002). The novel targeted drug, ruxolitinib, has only been employed in limited cases, with varied responses (Szuber et al., 2020). Moreover, prognostic biomarkers for advanced CNL patients and targeted therapy are markedly lacking. Consequently, the survival heterogeneity continues to perplex clinicians.

**FIGURE 1:**
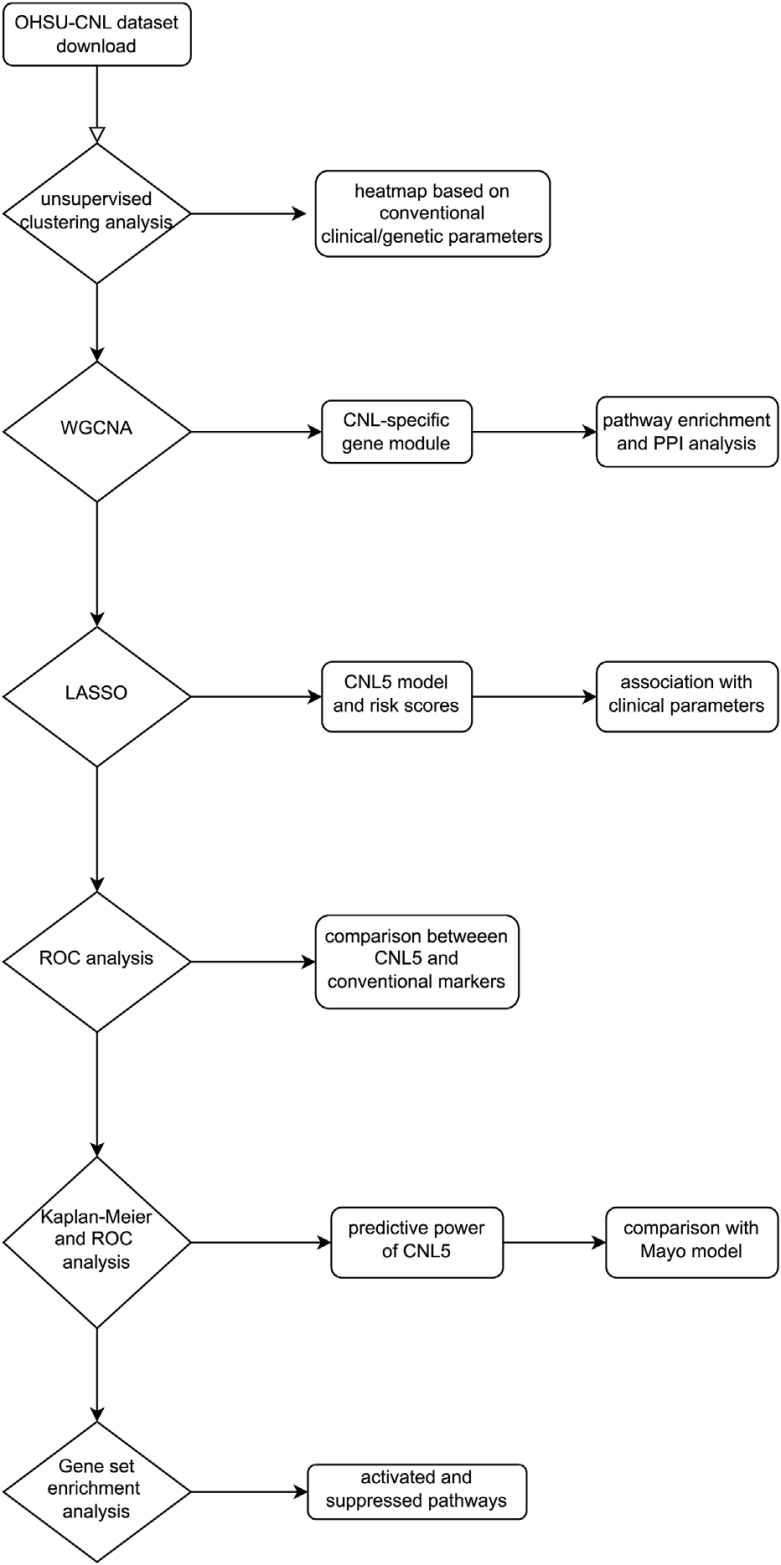
Research design flowchart of this study.

The largest cohort for transcriptomic exploration of rare neutrophilic leukemias was executed by Prof. Tyner and his collaborators (Zhang et al., 2019), incorporating CNL, aCML, CMML, etc. Emerging methodologies have identified key genes and pathways based on RNA data, where the weighted gene co-expression network analysis (WGCNA) based on scale-free network theory has examined the interplay of co-expressing gene clusters. The entire genome was partitioned into a selected number of modules, each containing genes co-expressed in individual samples. Subsequently, a correlation analysis was performed between clinical/genetic traits (e.g., diagnosis and symptoms) and module eigengenes (MEs, defined as the primary principal component of gene co-expression modules) (Langfelder and Horvath, 2008; Zhang and Horvath, 2005). This analysis resulted in a co-expression module most pertinent to CNL. Ultimately, a groundbreaking transcriptomic diagnostic model was developed employing the least absolute shrinkage and selection operator (LASSO). The flowchart illustrating the study is shown in Figure 1.

## Materials and methods

### Data download

The OHSU-CNL datasets were procured from the Genomic Data Commons (GDC) database via the TCGAbiolinks package and R software (version 4.2.1). The OHSU-CNL dataset encompassed a total of 180 samples, with 41 CNL, 28 aCML, 30 CMML, 14 MDS/MPN-U, 13 MPN-U, 50 nebulous neutrophilic disorder, and four healthy donors. The distinct attributes of the clinical/genetic variables of the different diseases are cataloged in Table 1.

**TABLE 1:** Summary of clinical and genetic variables in the OHSU-CNL datasets. RBC, red blood cell; WBC, white blood cell.

Clinical/genetic variables	CNL	aCML	CMML	MDS/MPN-U	MPN-U
Age	68.4 ± 12.5	69.3 ± 12.1	68.4 ± 11.6	68.1 ± 11.0	64.3 ± 18.9
Gender (female/male)	17/24	6/22	11/19	4/10	8/5
Splenomegaly	28/34	20/27	10/24	12/13	3/8
Leukemic transformation	6/31	4/23	6/29	4/12	2/10
Major bleeding	5/38	2/24	0/29	2/13	3/11
Thrombosis	2/38	2/26	2/29	1/13	0/11
Hemoglobin (g/dL)	11.6 ± 2.09	10.0 ± 2.16	11.0 ± 2.64	10.7 ± 1.76	10.7 ± 2.63
RBC transfusion	19/37	18/25	14/30	12/13	7/11
Transfusion need	10/38	11/23	12/30	4/13	4/10
WBC (×10^9^/L)	62.1 ± 59.4	77.3 ± 66.3	37.0 ± 41.2	44.5 ± 33.7	81.6 ± 95.2
Blasts (%)	0.621 ± 1.78	1.79 ± 2.65	1.63 ± 2.92	0.889 ± 2.13	1.10 ± 2.02
Immature granulocytes (%)	2.86 ± 7.80	18.92 ± 13.3	5.45 ± 4.77	9.67 ± 8.07	14.3 ± 15.1
Neutrophils (%)	84.9 ± 10.1	67.4 ± 17.5	53.9 ± 16.0	67.5 ± 15.9	65.6 ± 15.9
Monocytes (%)	2.66 ± 3.52	2.76 ± 3.48	19.9 ± 11.4	5.63 ± 6.70	4.00 ± 1.61
BM cellularity	91.7 ± 8.89	91.4 ± 9.76	83.0 ± 16.1	91.5 ± 5.80	84.4 ± 17.2
BM dysplasia	12/35	20/26	17/25	14/14	0/9
BM dysgranulopoiesis	2/32	10/23	2/13	2/9	0/9
CSF3R mutation	25/39	6/27	1/29	1/12	0/9
Survival (days)	879 ± 827	731 ± 920	895 ± 732	745 ± 981	594 ± 374

To validate the diagnostic power, the MDS/MPN part of the GSE42731 dataset was also downloaded from the Gene Expression Omnibus (GEO) database, and it included seven CMML, four MPN, two CML, two PMF, two CNL, one JMML, and one ET.

### Clustering analysis based on conventional clinical/genetic variables

A total of 120 samples had parallel clinical/genetic information (diagnosis, hemoglobin, white blood cell count, blast percentage, immature neutrophil percentage, monocyte percentage peripheral neutrophil percentage, bone marrow cellularity, dysplasia, dysgranulocytosis, leukemic transformation, thrombosis, major bleeding, red blood cell transfusion, transfusion need, splenomegaly, CSF3R, and other gene mutations). Subsequently, employing the k-means method, these 120 samples underwent unsupervised clustering analysis, thereby partitioning them into closely related groups based on clinical/genetic variables to validate whether neutrophilic leukemias could be differentiated.

### WGCNA

Seventy-five out of 120 patients in the OHSU-CNL cohort, with sufficient clinical/genetic and transcriptomic data, were inputted in the subsequent WGCNA analysis. The WGCNA package of the R software (version 4.0.2) was employed for co-expression analysis (Langfelder and Horvath, 2008). Inter-individual heterogeneity was calculated via the hierarchical clustering method using average linkage to identify and exclude outliers. The minimum beta value yielding a scale-free R^2^ greater than 0.85 was determined to be the soft-threshold power. Pearson’s coefficients between individual genes were calculated, forming the basis for establishing the gene adjacency matrix and the topological overlap matrix. Guided by hierarchical clustering using average linkage, the entire genome was segmented into gene co-expressed modules with over 25% inter-module dissimilarity. Pearson’s coefficients between module eigengenes (MEs) and expression values of individual intra-module genes were denoted as module membership (MM). Pearson’s coefficients between trait and expression values of individual intra-module genes were designated as gene significance (GS). The module with the highest Pearson’s coefficient with CNL and statistically irrelevant or negatively correlated with other neutrophilic leukemias was identified as the CNL-specific module. Intra-module genes with a GS ≥ 0.2, MM ≥ 0.8, and weighted q value <0.01 were classified as hub genes.

Sample 3079R showed significant heterogeneity compared to the other patients and was consequently excluded from subsequent analyses (Supplementary Figure 1). A total of 74 patients were incorporated into the WGCNA: 15 with CNL, 18 with aCML, three with CMML, seven with MDS/MPN-U, five with MPN-U, and 26 with ambiguous neutrophilic disorders. By considering the scale-free R^2^ distribution (Supplementary Figure 2), a soft-threshold power of 9 was chosen. The entire gene set was partitioned into 39 gene modules with less than 25% dissimilarity (Supplementary Figure 3). To discern the correlations between these modules, a heatmap was created to visualize topological overlap and module eigengene adjacency based on 400 randomly selected genes from different modules (Supplementary Figure 4).

### Pathway enrichment and protein–protein interaction (PPI) network analysis

The Kyoto Encyclopedia of Genes and Genomes (KEGG) database was employed to delineate the spectrum of pathways enriched by the CNL-specific module (Huang da et al., 2009). Pathways bearing a local false discovery rate (FDR) adjusted p-value of less than 0.05 were deemed significant.

Bolstered by extant evidence, the hub genes of the CNL-specific module were integrated into the STRING database (https://string-db.org/) (Szklarczyk et al., 2019) to construct a protein–protein interaction (PPI) network. Protein–protein pairs with an interaction score exceeding 0.4 were harvested as edges of the PPI network. Utilizing Cytoscape software (version 3.7.2) along with the CytoHubba plugin, the connectivity of nodes was calculated and ranked, thereby facilitating the selection of central nodes.

### Establishment of novel diagnostic models

The brown gene module expression data from 53 patients with non-missing diagnoses were inputted into LASSO analysis after normalization of the raw count data using the varianceStabilizingTransformation () function and removal of missing values using the na.omit () function. Then, we implemented LASSO regression using the glmnet package (version 4.1–8) in the R software. The optimal lambda value was selected through 10-fold cross-validation (using the cv.glmnet () function with “unfolds = 10”), and lambda.min was used as the final regularization parameter for the model. Model performance was evaluated using mean squared error (MSE) and R^2^. After iterating LASSO 10,000 times to curtail overfitting, variables with non-zero coefficients were identified. Subsequently, the bootstrap aggregation method gave rise to a model consisting of an expression signature of five genes (PDCD7, CR2, ZSCAN70, TRIM68, and LILRA6), referred to as the CNL-5 model. The CNL-5 risk scores for individual patients were calculated by summing the expression values of selected genes, each weighted by their corresponding coefficients.

### Clinical relevance of the CNL-5 model

Patients (n = 74) possessing expression information were divided into CNL-like and atypical-CNL groups, dichotomized by the ROC-defined cut-off value. Subsequently, the clinical/genetic parameters of the different groups were compared, which included age, gender, splenomegaly, major bleeding, thrombosis, hemoglobin, RBC transfusion, platelet transfusion need, WBC, blast percentage, immature granulocyte percentage, neutrophil percentage, monocyte percentage, bone marrow cellularity, dysplasia, dysgranulopoiesis, and mutant CSF3R.

### Comparison of the novel CNL-5 risk scores with conventional clinical/genetic markers

Established diagnostic indicators for CNL include an elevated percentage of neutrophils and a CSF3R mutation. Thus, the clinical utility of our novel models was elucidated through their comparison with these conventional markers, employing multiple ROC analyses and AUC estimation via the GraphPad Prism (version 9.0) software. Validation of the CNL-5 model was performed on the GSE42731 dataset using ROC analysis on the diagnostic utility.

### Survival analysis for the CNL-5 model

A selection of 46 patients (with 12 CNL, 17 aCML, three CMML, seven MDS/MPN-U, three MPN-U, and four ambiguous neutrophilic disorder cases) with both expression data and overall survival was included to substantiate the prognostic value of the CNL-5 model. The surv_cutpoint function from the survminer package was employed to compute the cut-off value, effectively segmenting these patients into CNL-5 high- or low-risk cohorts. The survival and survivalROC packages were utilized to conduct the Kaplan–Meier analysis and time-dependent ROC. A Mayo Clinic risk model for CNL (Szuber et al., 2018) was used as the control to compare the predictive power of the Kaplan–Meier plots.

### Gene set enrichment analysis

Pearson’s coefficients were computed to assess the correlation between CNL-5 risk scores and individual gene expression values. We utilized gene set enrichment analysis (GSEA) to decipher the results of the genome-wide correlation analysis for CNL-5 risk scores, based on the coefficients of genes in the specific sets (pathways) of the Molecular Signatures Database (MSigDB) (http://software.broadinstitute.org/gsea/msigdb) (Subramanian et al., 2005). Given *a priori*-defined gene sets (such as oxidative phosphorylation, etc.), genes within a specific set were ranked by their Pearson’s coefficient with CNL-5 risk scores. Concurrently, the q value was employed to correct for multiple hypothesis testing. GSEA, which is based on the transcript level of all genes within a specific pathway rather than a few regulators, offered a more comprehensive depiction of the activation/suppression profile of signaling pathways associated with CNL-5 risk scores. Signaling pathways with an absolute normalized enrichment score (|NES|) greater than 1 and a q value less than 0.05 were deemed significantly associated with CNL-5 risk scores.

### Statistical analysis

If the continuous variables of the subgroups conformed to a normal distribution, the unpaired t-test was used to compare different groups. Otherwise, the non-parametric Mann–Whitney test was implemented. An ordinary one-way ANOVA test was applied to compare three or more subgroups of variables. The two-sided Fisher’s exact test was used for categorical variables. All statistical tests were carried out assuming a significance level of p < 0.05 unless otherwise specified.

### Data sharing statement

The data supporting the findings of this study are available from the GDC database (https://portal.gdc.cancer.gov/) and the GEO database (https://www.ncbi.nlm.nih.gov/geo/), all of which are publicly available.

## Results

### Clustering analysis using clinical/genetic markers

Using the available clinical and genetic markers, the participants were stratified into five distinct groups utilizing the k-means clustering method (Supplementary Figure 5). Remarkably, each group encompassed a minimum of four different disease types, underscoring the inherent complexity and potential for misclassification in the diagnosis of neutrophilic leukemias when relying solely on current parameters.

### Results of the WGCNA

**FIGURE 2:**
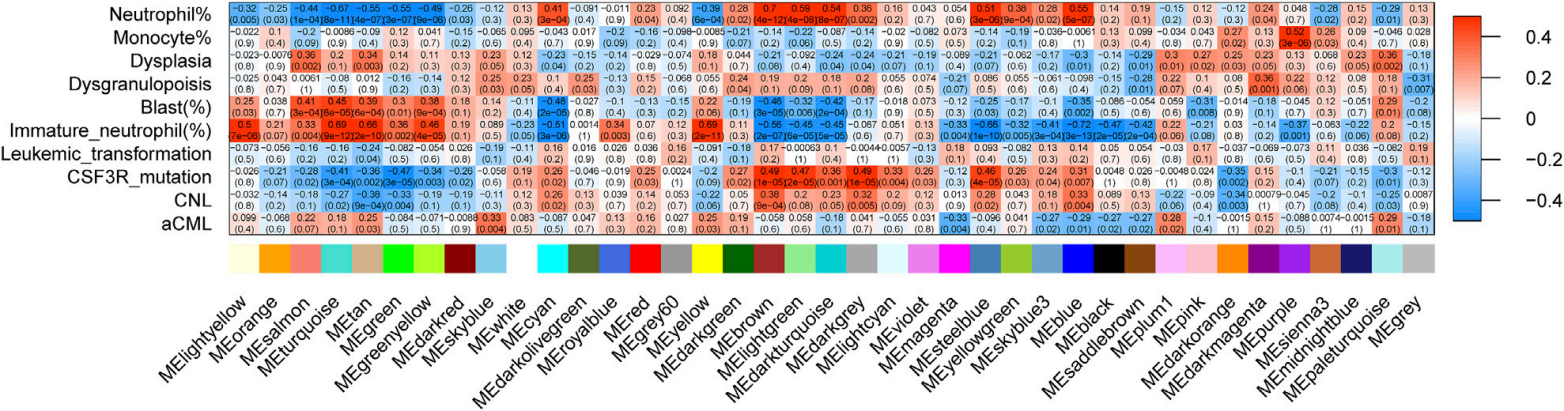
The relationship between gene co-expression clusters and disease subtypes. Each box contains Pearson’s coefficients (by color gradient, red = 1, blue = −1) and p-values for the correlation between MEs (X-axis) and clinical/genetic variables (Y-axis).

The correlation between the modules and clinical/genetic traits, such as neutrophil percentage, monocyte percentage, dysplasia, dysgranulopoiesis, blast percentage, immature neutrophil percentage, leukemic transformation, disease types, and CSF3R mutation, is depicted in Figure 2. The brown module emerged as the CNL-specific module (R^2^ = 0.38, p = 9e−4; the detailed list of included genes can be found in Supplementary Table 1). Notably, the brown module displayed a significant correlation with the neutrophil percentage (R2 = 0.70, p = 4e−12) and CSF3R mutation (R2 = 0.49, p = 1e−5), both of which are primary clinical/genetic features of CNL. The brown module was not related to aCML (p > 0.05) and exhibited a negative correlation with the percentage of immature neutrophils and blasts (Figure 2). For the brown module, Supplementary Figure 6 shows the correlation of module membership (MM) and gene significance (GS) for the 152 intra-module genes, which suggests a significant association between the module eigengene and CNL.

### Pathway enrichment and PPI analysis

**FIGURE 3:**
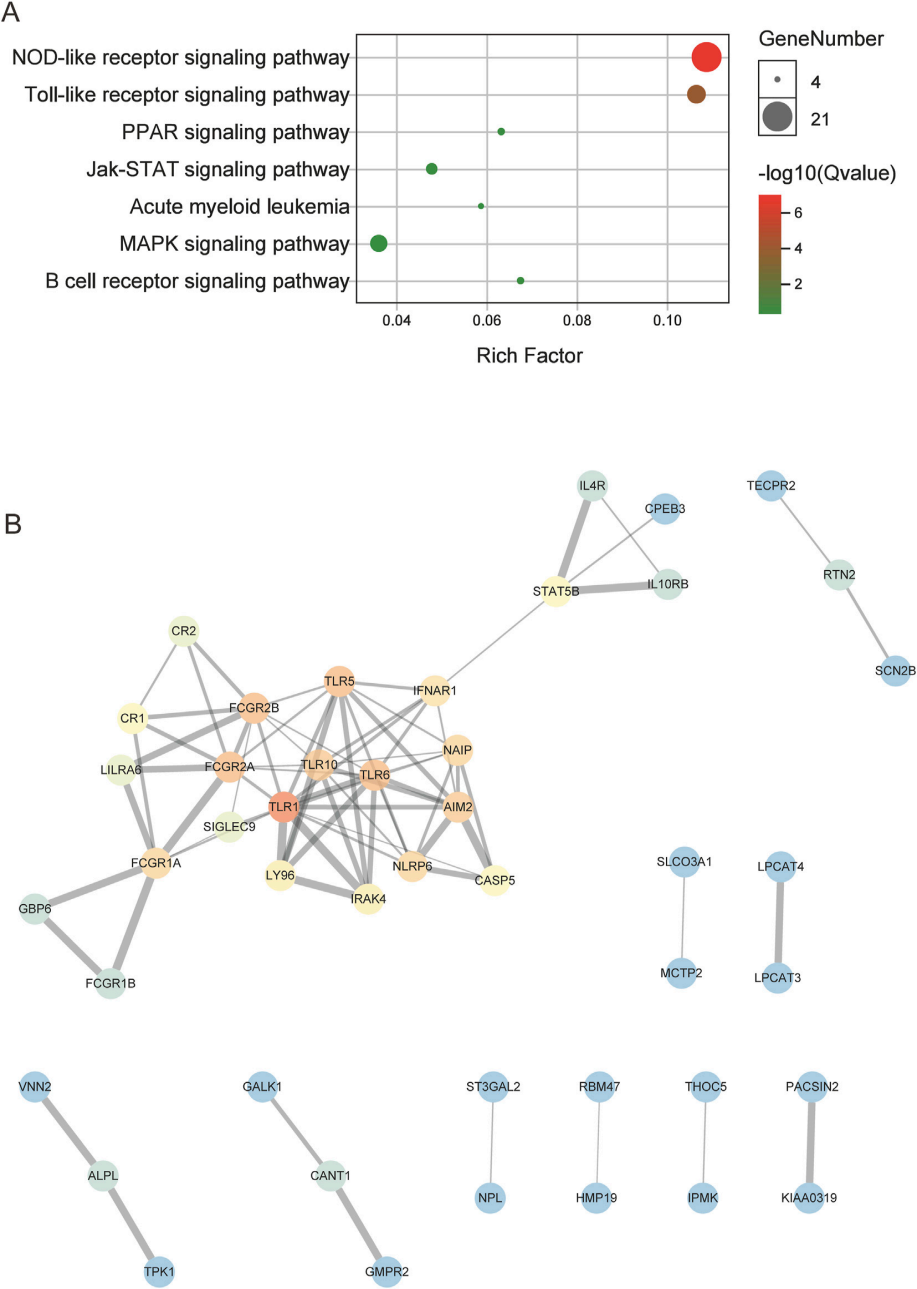
The results of enrichment and PPI analysis for the CNL-specific gene module. **(A)** The dot plot of enriched pathways. **(B)** The PPI network of hub genes within the CNL-specific module, in which the color gradient of the dots correlates with the connectivity degrees of individual genes (red for high degrees, blue for low degrees).

The hub genes in the CNL-specific module were found to be primarily enriched in pathways such as NOD-like receptor signaling, Toll-like receptor (TLR) signaling, and JAK-STAT signaling (Figure 3A). The topological structure of the PPI network, containing 116 nodes and 78 edges, is illustrated in Figure 3B. The top five genes with the highest degree of connectivity were TLR1, TLR5, TLR6, FCGR2A, and FCGR2B.

### The CNL-5 diagnostic model based on transcriptomic features

**FIGURE 4:**
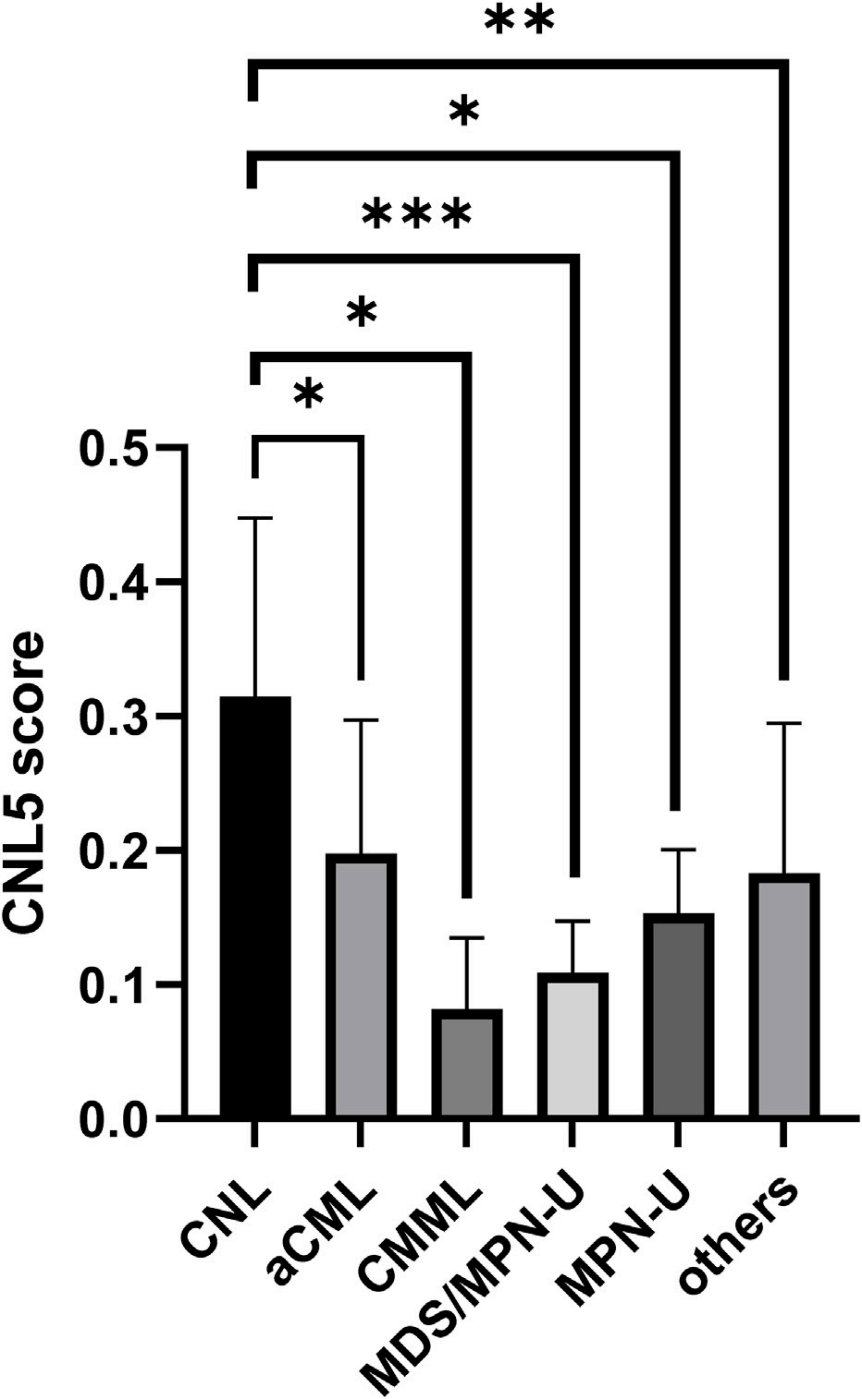
The bar plot comparing CNL-5 risk scores in CNL with other neutrophilic leukemias (*p < 0.05, **p < 0.01, and ***p < 0.001).

The variables with non-zero coefficients are displayed in Table 2. The CNL-5 risk scores of the samples were significantly higher in CNL than in other neutrophilic leukemias (Figure 4, p = 1.7e−5). A total of 74 patients were divided into CNL-like (n = 31) and Atypical-CNL groups (n = 43) based on the ROC-defined cut-off value (0.2044, Figure 5). Clinical and genetic variables were compared between the two groups, respectively (Table 3). The CNL-like group showed significantly superior erythroid/megakaryocytic hematopoiesis, receiving fewer RBC transfusions and less platelet transfusion need (p = 0.0053 and 0.0369, respectively) than the Atypical-CNL group. Furthermore, the patients in the CNL-like group displayed more typical clinical and genetic features (significantly lower blast and immature neutrophils percentage; higher peripheral neutrophil percentage and CSF3R mutation rate). Consequently, patients with neutrophilic leukemia and higher CNL-5 risk scores demonstrated clinical/genetic phenotypes more closely resembling those of classic CNL.

**TABLE 2:** Inclusion of gene symbols and corresponding coefficients for CNL risk models.

Gene symbol	Coefficient
PDCD7	−0.0813
CR2	0.00945
ZSCAN20	−0.00612
TRIM68	−0.0319
LILRA6	0.0196

**FIGURE 5:**
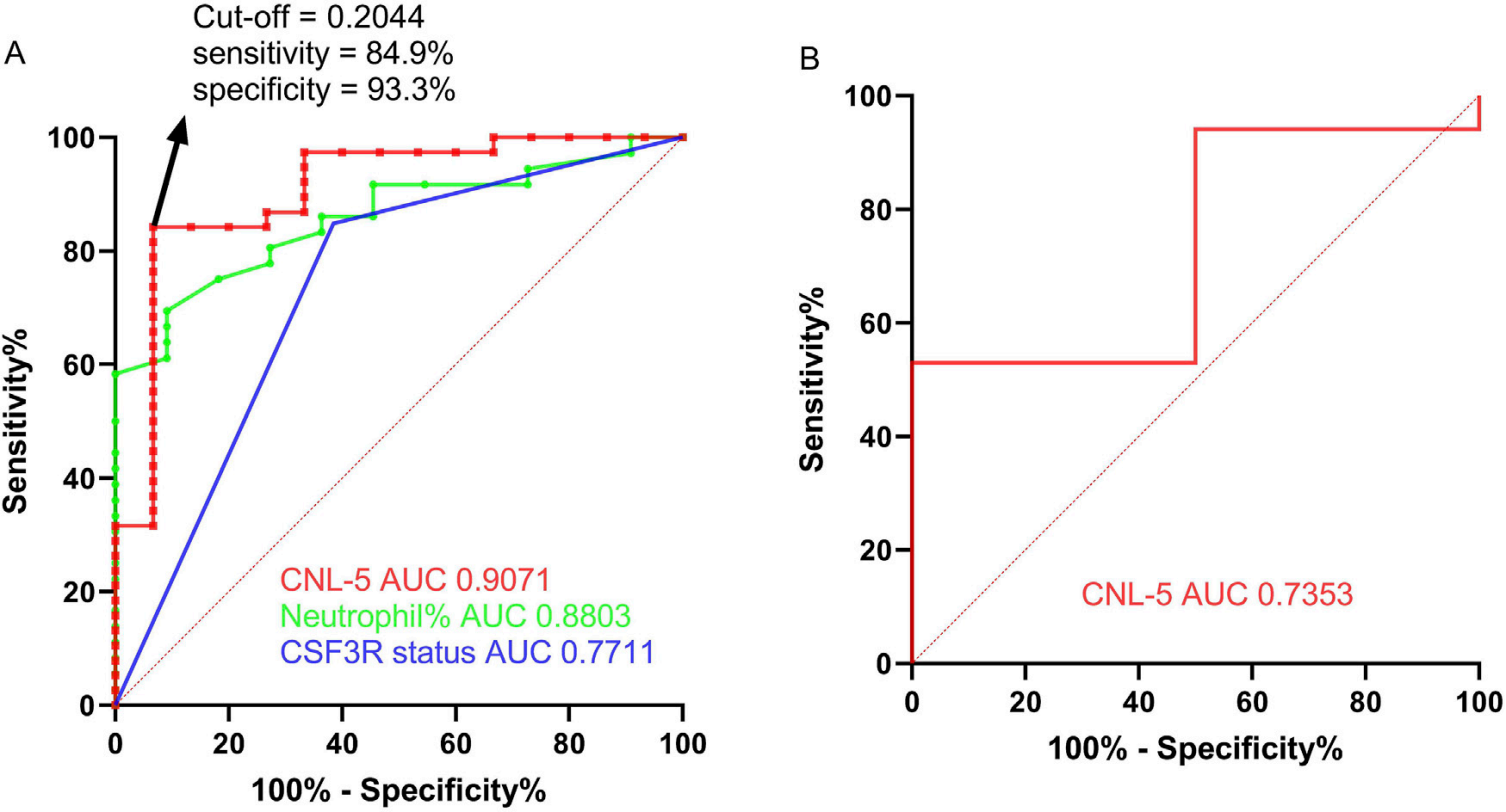
The ROC curve of the CNL-5 risk score in comparison with neutrophil percentage and CSF3R mutation status based on the OHSU-CNL cohort **(A)**. The ROC curve of the CNL-5 risk score is based on GSE42731 **(B)**.

**TABLE 3:** Comparison of clinical/genetic parameters between CNL risk groups.

	CNL-like	Atypical-CNL	p-value
Median age	74	70	0.1426
Gender (female/male)	11/20	14/30	0.8061
Splenomegaly	16/18	20/28	0.2736
Leukemic transformation	4/17	5/28	0.6447
Major bleeding	2/20	3/30	>0.9999
Thrombosis	2/20	2/30	0.6704
Median hemoglobin (g/dL)	10.4	10.6	0.9077
RBC transfusion	7/19	23/30	0.0053
Platelet transfusion need	3/19	13/29	0.0369
Median WBC (×10^9^/L)	54	47.7	0.857
Median blasts (%)	0	1	0.0123
Median Immature granulocytes (%)	0.5	13	0.0009
Median neutrophils (%)	86.5	63.5	<0.001
Median monocytes (%)	3.25	3.25	0.2423
Median BM cellularity	95	95	0.532
BM dysplasia	9/20	18/28	0.1842
BM dysgranulopoiesis	6/17	2/20	0.0625
Mutant CSF3R	12/26	1/34	<0.0001

### Comparison of diagnostic utility of CNL risk models with conventional markers

For the differential diagnosis of CNL from all other neutrophilic leukemias (aCML, MDS/MPN-U, MPN-U, and ambiguous neutrophilic disorder), the CNL-5 risk model demonstrated a superior AUC (0.9071, p < 0.0001) compared to CSF3R mutation status (AUC = 0.7711, p = 0.0152) and peripheral neutrophil% (AUC = 0. 8803, p = 0.0004) (Figure 5A). The confusion matrices are listed in Table 4. Moreover, when differentiating CNL from other types of neutrophilic leukemias, the CNL-5-risk model demonstrated a robust advantage over conventional markers (Supplementary Figure 7). This improvement has the potential to significantly enhance the precision of differential diagnosis in clinical practice. The predictive power AUC (0.7353, p = 0.05 in Figure 5B, confusion matrices in Table 4) was validated by the GSE42731 cohort under the stratification of the CNL-5 model.

**TABLE 4:** Confusion matrices of prediction by CNL5 model/neutrophil percentage/CSF3R status.

		OHSU-CNL cohort		GSE42731 cohort				OHSU-CNL cohort				OHSU-CNL cohort	
		CNL	Others	CNL	Others			CNL	Others			CNL	Others
CNL5 model	CNL-like	14	6	2	0	neutrophil percentage	>75.05%	10	13	CSF3R mutation	mutant	7	6
	Atypical-CNL	1	32	8	9		<75.05%	1	23		wild-type	5	28

### Prognostic value of the CNL-5 model

**FIGURE 6:**
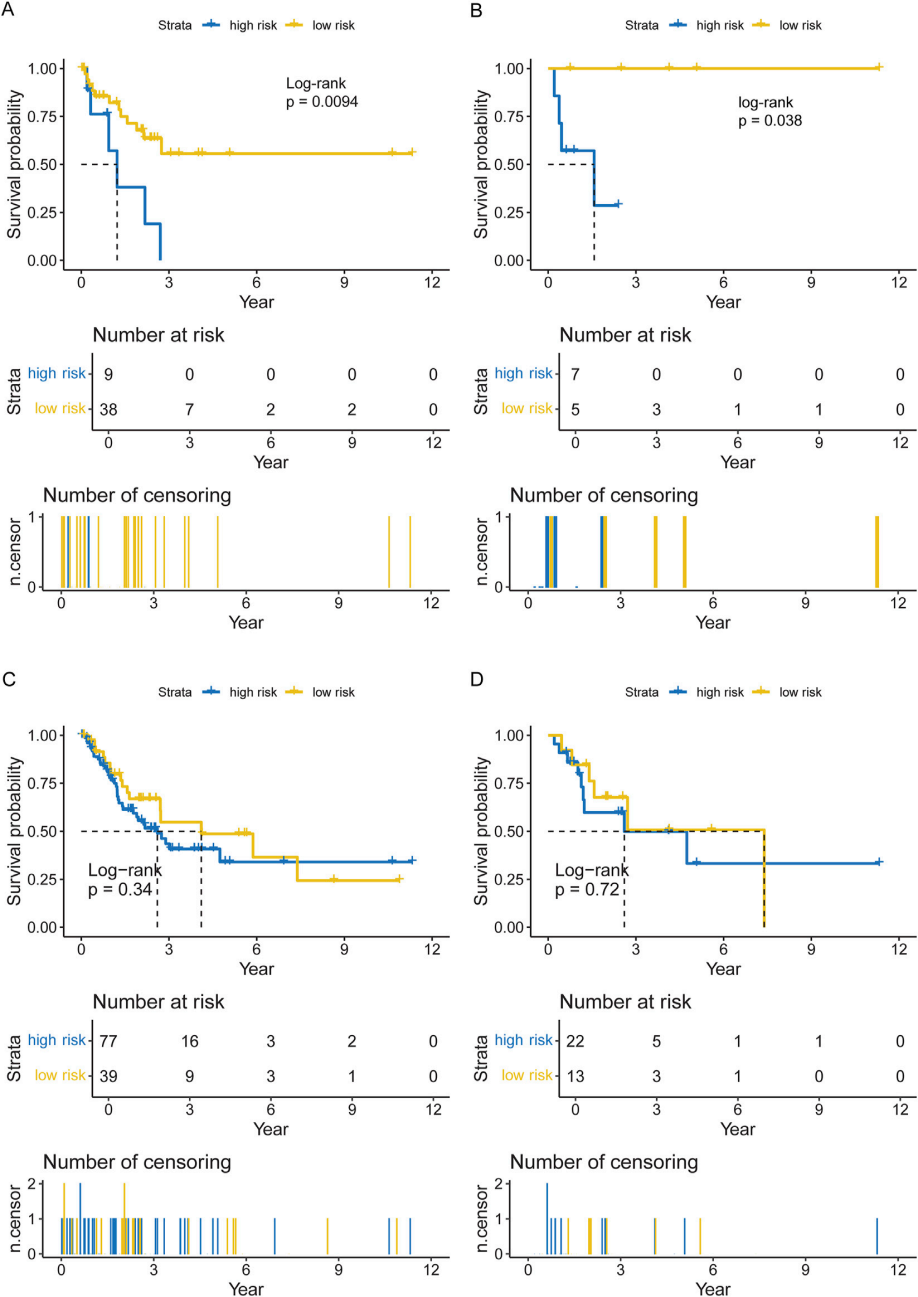
The Kaplan–Meier plots for overall survival of the CNL-5 high- and low-risk groups in the overall neutrophilic leukemia cohort **(A)** and the CNL subset **(B)**; and the Kaplan–Meier plots for overall survival of the Mayo Clinic high- and low-risk groups in the overall neutrophilic leukemia cohort **(C)** and the CNL subset **(D)**.

**FIGURE 7:**
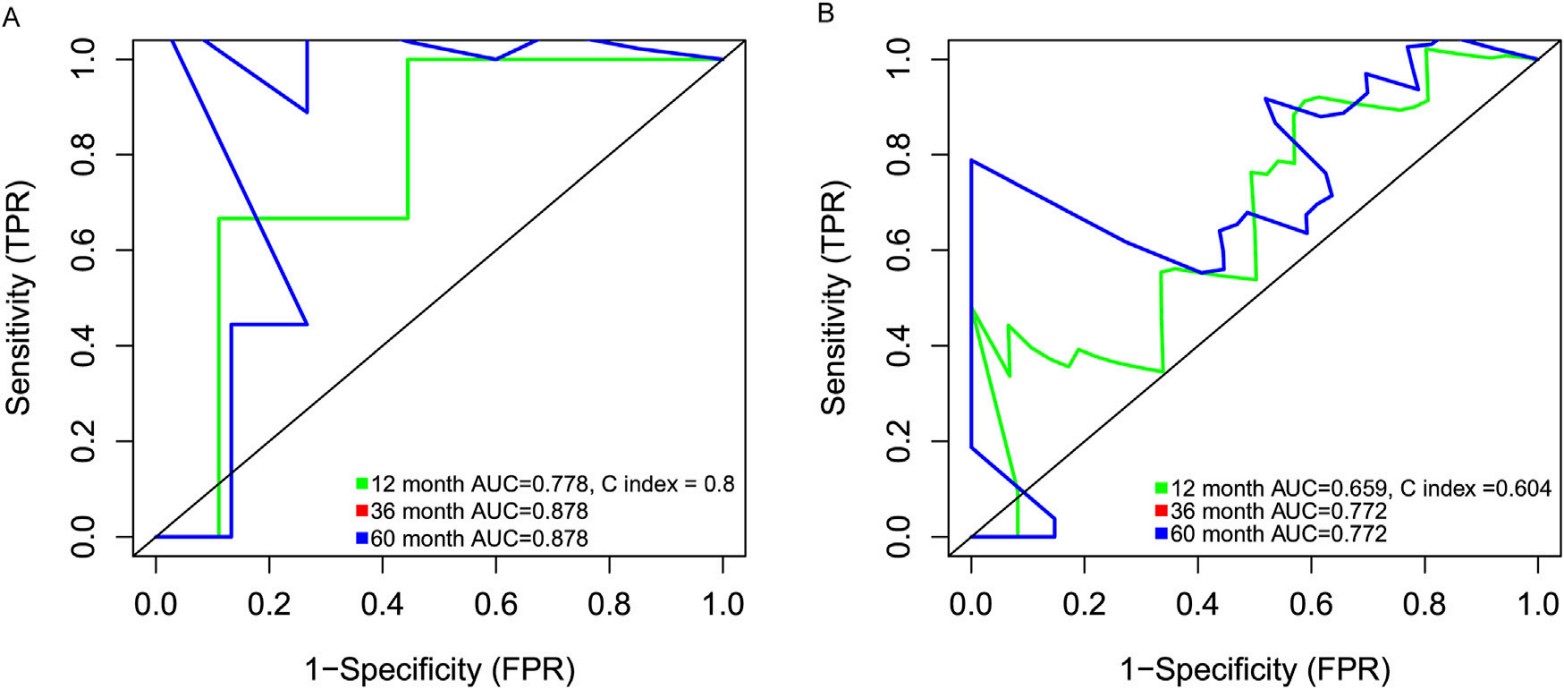
The time-dependent ROC analysis deciphering the AUC and Harrison’s C index of the CNL5 model in the CNL subset **(A)** and the overall neutrophilic leukemia cohort **(B)**.

Data from 47 neutrophilic leukemia patients in the OHSU-CNL cohort with sufficient overall survival data were inputted into the survival analysis. The cut-off value based on the CNL-5 model was 0.1, which divided the 47 patients with neutrophilic leukemias into high and low-risk groups (n = 9 and n = 38, respectively). The overall survival of the high-risk group was significantly worse than that of the low-risk group (Figure 6A, log-rank p = 0.0094). A similar result was seen in the CNL subset, where all low-risk patients attained long-term survival (Figure 6B, log-rank p = 0.038). A Mayo Clinic-approved risk model for CNL (Szuber et al., 2018) was tested in the OHSU-CNL cohort by Kaplan–Meier analysis, while no significant survival discrimination was revealed in both the whole neutrophilic leukemia cohort and the subset of CNL patients (Figures 6C, D). Time-dependent ROC analysis demonstrated that the CNL-5 model provided prognostic value, AUC, and Harrison’s C index for both the CNL subset and the whole neutrophilic leukemia cohort (Figures 7A, B).

### Whole-transcriptome correlation analysis of CNL-5 risk scores

**FIGURE 8:**
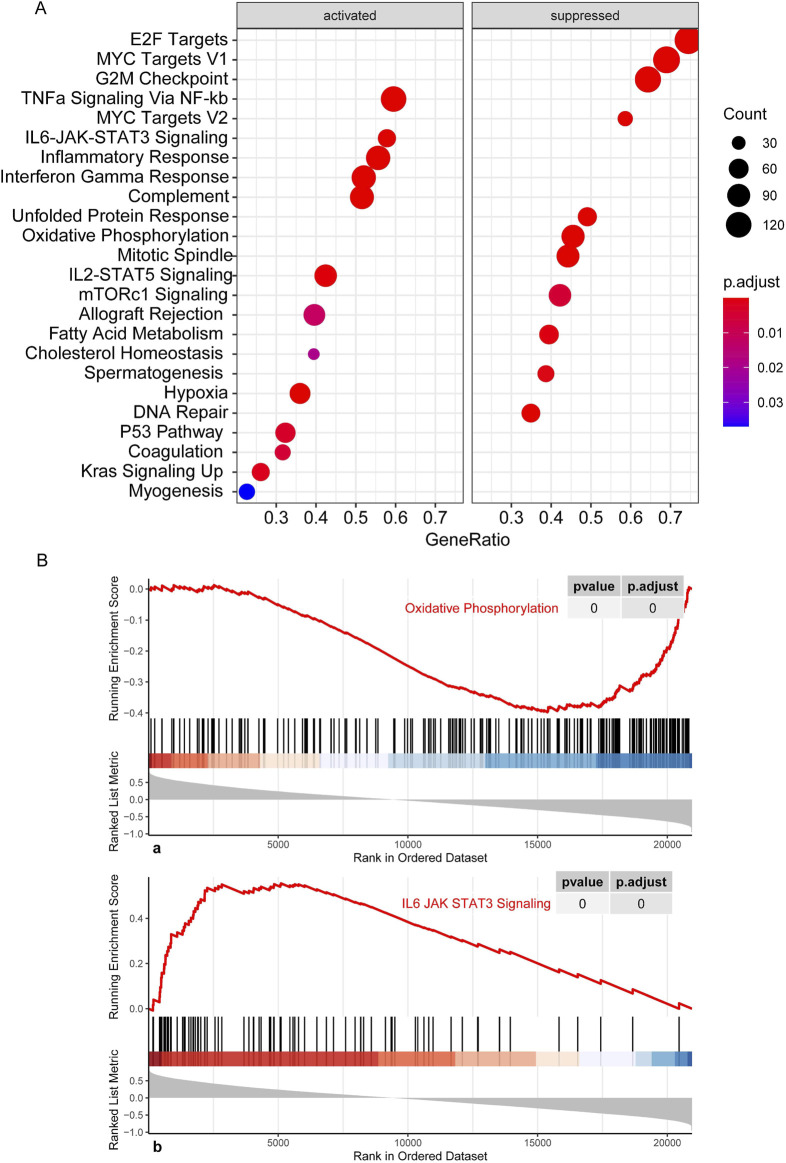
GSEA results showing the activation/suppression profile of cell signaling pathways **(A)**. Running enrichment score curve for pathways significantly correlating with CNL-5 risk scores **(B)**. The x-axis represents the genes in the whole genome, and the bars correspond to specific genes in the pre-defined sets. The most positively correlated genes are listed to the left of the whole genome axis, while the most negatively correlated genes are listed to the right. The enrichment score (red line) represents a running-sum statistic that increased when we encountered a gene in the specific set and decreased when we encountered genes not in the specific gene set. The magnitude of the increment depends on the correlation of the gene with the CNL5 risk scores: the location of the maximum enrichment score is on the left part of the x-axis, and the normalized enrichment scores (NES) are positive.

CNL-5 risk scores were correlated with suppression of oxidative phosphorylation and activation of IL6-JAK-STAT3 signaling, P53 signaling, and upregulation of Kras (Figure 8), etc. To investigate the JAK-STATs signaling, the association of CNL-5 risk scores with the expression level of individual JAKs (JAK1-3 and TYK2) and STATs (STAT1-4, STAT5A/B, and STAT6) was investigated. The results indicated that there was a positive correlation between CNL-5 risk scores and JAK2, JAK3, TYK2, STAT4, STAT5B, and STAT6 (Supplementary Figure 8) and a negative correlation with STAT2.

## Discussion

Conventional clinical/genetic markers have been insufficient to classify neutrophilic leukemias accurately thus far (Supplementary Figure 5). Moreover, previous investigations on mutation profiles have not fully elucidated the heterogeneity within neutrophilic leukemias. No specific mutation pattern was found.

A recent preliminary study revealed that CNL and aCML differed in gene expression profiles based on 172-gene target sequencing (Sun et al., 2024). To further explore the transcriptome for this group of rare myeloid diseases, we aim to extend the gene expression spectrum to the whole genome and the disease spectrum to ambiguous neutrophilic leukemias. In the original study on the OHSU-CNL cohort (Zhang et al., 2019), WGCNA was also implemented to divide the whole genome into nine gene mods and analyze the relationship between gene mods with clinical variables (age, WBC, survival, etc.). Based on the previous analysis, we used WGCNA to detect the gene co-expression module corresponding to CNL specifically (Figure 2, brown module). Constitutive JAK-STAT signaling resulted from activating mutations of CSF3R in the extracellular (T618I) or transcellular (T615A) domains, which occur more frequently in CNL than in aCML (Maxson et al., 2014; Maxson et al., 2013; Pardanani et al., 2013). Consistently, brown module genes were enriched in JAK-STAT signaling pathways (Figure 3), which validated the accuracy of our WGCNA analysis.

Then, brown module genes were inputted into LASSO to establish a diagnostic model with the optimal AUC through sufficient cycles of iterations. Intriguingly, it was noted that not all patients in the CNL-like group carried mutant CSF3R (12 out of 26 with wild-type CSF3R, Table 3), which may change future disease subtyping and/or treatment strategy for neutrophilic leukemia patients with a CNL-like expression signature instead of CSF3R mutations. Meanwhile, in the aCNL group, one aCML patient had CSF3R gene mutations (missense + truncation), suggesting the transcriptomic heterogeneity in CSF3R-mutant patients. The CNL-5 model was associated with a CNL-like phenotype (Table 3) and had a significantly better predictive power (AUC) than that of conventional parameters in discriminating CNL from other neutrophilic leukemias (Figure 5; Supplementary Figure 6). The robustness of the predictive power was validated by an external cohort (GSE42731) with a lower AUC. The difference in AUC between the OHSU-CNL and the GSE42731 cohorts may have resulted from the respective methodologies (RNAseq vs. microarray) and unbalanced disease distribution with only two CNL patients of the 19 in GSE42731.

This model will help clinicians to diagnose CNL based on the transcriptomic signature in combination with clinical/genetic parameters. Moreover, the CNL-5 risk model also predicted the survival of patients with neutrophilic leukemias, not only in the CNL subset but neutrophilic leukemias in general (Figures 6, 7). The Mayo Clinic model, which incorporated clinical parameters and ASXL1 status, failed to stratify survival in either situation. This result will help physicians to identify patients with dismal outcomes and provide more radical therapies (such as all-HSCT, etc.).

The five genes (Table 2) of the CNL-5-risk model have been rarely investigated in myeloid neoplasms, including PDCD7 (programmed cell death protein 7), CR2 (complement receptor type 2), ZSCAN20 (zinc finger and SCAN domain-containing protein 20), TRIM68 (E3 ubiquitin-protein ligase TRIM68), and LILRA6 (leukocyte immunoglobulin-like receptor subfamily A member 6). Functional experiments are still needed to validate the pathological role of the five genes.

Little was known about the cell signaling profile in chronic neutrophilic leukemia cells, and this study provided some insights. Based on GSEA, the CNL-5 risk scores were associated with activation of the cytokine-JAK-STAT pathway, such as IL6-JAK-STAT3 and IL2-STAT5 signaling (Figures A, B). JAK inhibitors, such as ruxolitinib, have been demonstrated to have varied efficacy against CNL *in vivo* and *in vitro* (Dao et al., 2020). The detailed mechanisms of JAK-STAT activation in CNL are still unclear because few samples are available from patients, and animal models for CNL are not established. The following correlation analysis revealed that CNL-5 risk scores were positively correlated with JAK2, JAK3, TYK2, STAT4, and STAT5B while negatively and significantly correlated with STAT2 (Supplementary Figure 8), suggesting a specific expression pattern of JAK-STAT signaling and the potential of combined JAK/TYK inhibition.

Inflammation-related pathways were also correlated with CNL-5 risk and enriched in the CNL-specific gene module (Figures 3, 8), such as NOD-like receptor signaling, Toll-like receptor signaling, inflammatory response, and the cytokine-JAK-STAT pathway. Because the inflammatory gene expression signature is associated with treatment response and clinical outcome (Gower et al., 2025; Stratmann et al., 2022; Mukherjee et al., 2022), the association of CNL-5 risk and leukemia-related inflammatory genes was also analyzed. IRF5 regulon genes, which have been reported to be associated with poor outcomes in T-ALL were significantly correlated with CNL-5 risk (Supplementary Figure 9). The IRF5 regulon signature indicated reduced sensitivity to dexamethasone and high sensitivity to venetoclax (Gower et al., 2025), which may provide insight into novel treatment choices for CNL, especially in patients with hyperleukocytosis. Another inflammatory gene signature, mainly involving IRF7/9 and STAT5B, which are associated with chemoresistance in refractory ETP-ALL (Jason et al., 2024-11), was also significantly relevant to CNL-5 risk scores (Supplementary Figure 8). Moreover, other than ALL, an inflammatory gene expression signature was reported to be related to AML disease progression (Stratmann et al., 2022) and could discriminate between *de novo*/secondary AML (Davis et al., 2024). Significant overexpression of IL1R1/INSR and underexpression of CR1 were correlated with CNL-5 risk, which was reported to be associated with AML progression (Stratmann et al., 2022) (Supplementary Figure 9).

The enrichment of significant overexpression on IL18RAP, IL18R1, IL1R1, IL2RB, and IL4R indicated that CNL resembled an inflammatory signature of secondary AML (Supplementary Figure 9). IL18R1 expression also correlates with inflammation and high NLR (neutrophil/lymphocyte ratio) in melanoma (Mallardo et al., 2023), which validated the prognostic role of the inflammatory gene signature in addition to hematopoietic malignancies. These results suggested that the dismal outcome in the CNL-5 high-risk group may be partly related to leukemia-promoting inflammatory environments.

Another intriguing point was the metabolism-related pathways associated with CNL-5-risk scores. The majority of the evidence indicated that the dominant method of obtaining ATP was glycolysis. In 2017, Riffelmacher et al. reported that the maturation of neutrophils requires a metabolic transition from glycolysis to oxidative phosphorylation fed by free fatty acids, resulting from ATG5/ATG7-dependent autophagy (Riffelmacher et al., 2017). Intriguingly, the expression of ATG5 and ATG7, essential autophagy genes, was positively correlated with CNL-5 risk scores (Supplementary Figure 10). CNL risk scores were associated with suppression of oxidative phosphorylation (OXPHOS) and fatty acid metabolism (Figures 7, 8). This paradoxical phenomenon, which enhanced ATG5/7 and attenuated OXPHOS, suggested heterogeneity of neutrophil function and an abnormal relationship between autophagy and OXPHOS in CNL. Further studies are needed to explore the detailed metabolic changes of neutrophils in neutrophilic leukemias compared to normally differentiated neutrophils.

Other signaling pathways had no evidence of association with neutrophils or CNL but may provide clues for investigation of therapeutic targets, such as Kras signaling, interferon, gamma response, etc.

One limitation of our study is the insufficient number of samples, predominantly due to the rarity of neutrophilic leukemias. The CNL-5 model still requires large-scale cohorts to be validated.

## Conclusion

In this study, a co-expression signature specific to CNL was identified among ambiguous neutrophilic leukemias. Based on this signature, a novel CNL-5 risk model was established that demonstrated better diagnostic utility than conventional markers. This study also provided valuable insights into the signaling pathway profiles and potential therapeutic targets for CNL.

## Data Availability

The data presented in the study are deposited in: the Genomic Data Commons (GDC) repository, accession number OHSU-CNL (https://portal.gdc.cancer.gov/); the Gene Expression Omnibus (GEO) repository, accession number GSE42731 (https://www.ncbi.nlm.nih.gov/geo/).

## Supplementary material

SUPPLEMENTARY FIGURE 1 The results of clustering the included CNL samples by the average linkage hierarchical clustering method.

SUPPLEMENTARY FIGURE 2 The value of scale independence (left) and mean connectivity (right) to identify the soft threshold in the following network analysis.

SUPPLEMENTARY FIGURE 3 The cluster dendrogram (top) and gene co-expressed modules (bottom), where the height of the branches represents the Euclidean distance.

SUPPLEMENTARY FIGURE 4 The module eigengene adjacency heatmap **(A)** indicates the relationship between distinct co-expression modules. The results of module clustering are shown in the upper part **(B)**.

SUPPLEMENTARY FIGURE 5 The results of sample clustering and heatmap for clinical/genetic parameters by k-mean clustering method. The X-axis represents the individual patients, and the Y-axis represents the clinical/genetic factors on the right side. The color of the dots indicates the status of the variables. For categorical variables, such as gene mutations or clinical events (thrombosis, major bleeding, etc.), red dots indicate positive results. Otherwise, the dots are in blue. For continuous variables, such as neutrophil percentage, etc., the redder dots represent higher values, and the bluer dots represent lower values. The gray dots indicate the missing value of the corresponding samples.

SUPPLEMENTARY FIGURE 6 Correlation of MM and GS in the brown module.

SUPPLEMENTARY FIGURE 7 The diagnostic ROC for AUC of CNL risk scores versus conventional markers for CNL versus aCML **(A)**; CNL versus CMML **(B)**; CNL versus MDS/MPN-U **(C)**; CNL versus MPN-U **(D)**.

SUPPLEMENTARY FIGURE 8 The correlation analysis between CNL-5 risk scores and JAKs/STATs.

SUPPLEMENTARY FIGURE 9 The correlation analysis between CNL-5 risk scores and IRF5 regulon genes.

SUPPLEMENTARY FIGURE 10 The correlation analysis between CNL risk scores and AML-related inflammatory gene expression signature.

SUPPLEMENTARY FIGURE 11 The correlation analysis between CNL risk scores and ATG5/7.

SUPPLEMENTARY TABLE 1 The list of hub genes in the CNL-specific (brown) module.
