# Supplementary figures and images for: Transcriptomic signature can distinguish chronic neutrophilic leukemia from ambiguous neutrophilic leukemias
Source: Front Genet. 2025 Apr 4;16:1556519. doi: 10.3389/fgene.2025.1556519 (PMC12006112; doi:10.3389/fgene.2025.1556519)

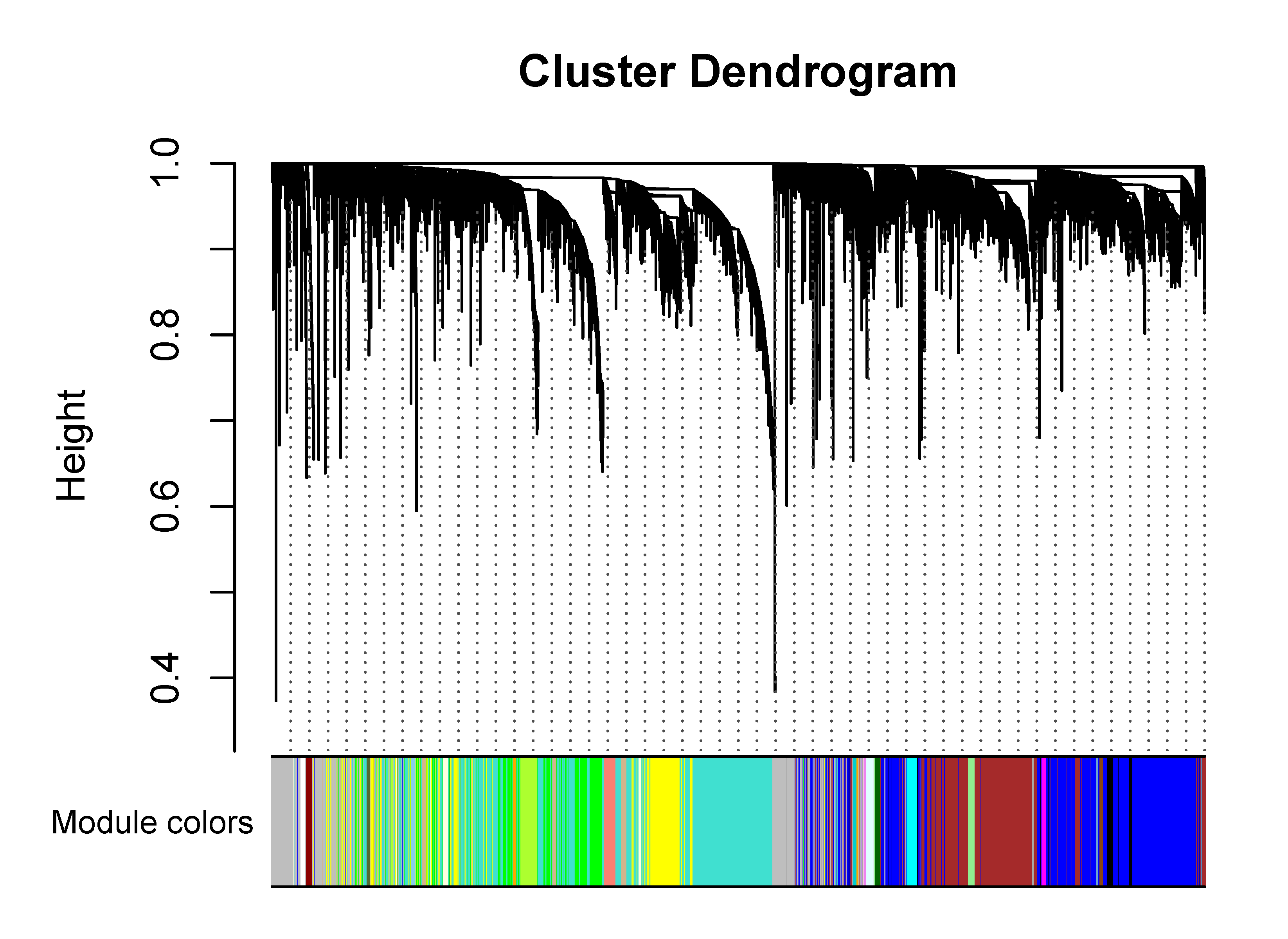

Supplement: Supplementary file 1 [file Image3.tiff]

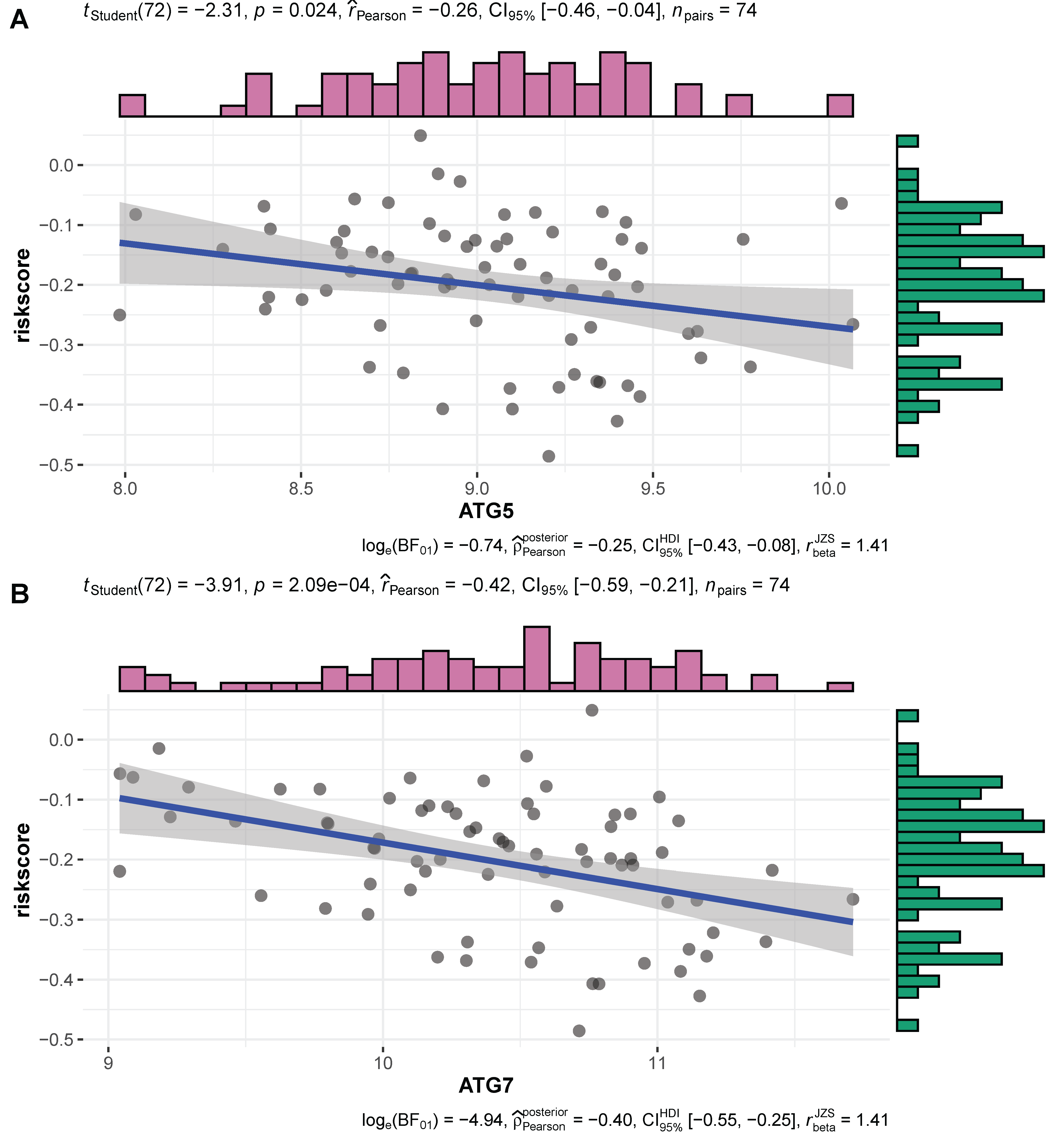

Supplement: Supplementary file 6 [file Image11.tiff]

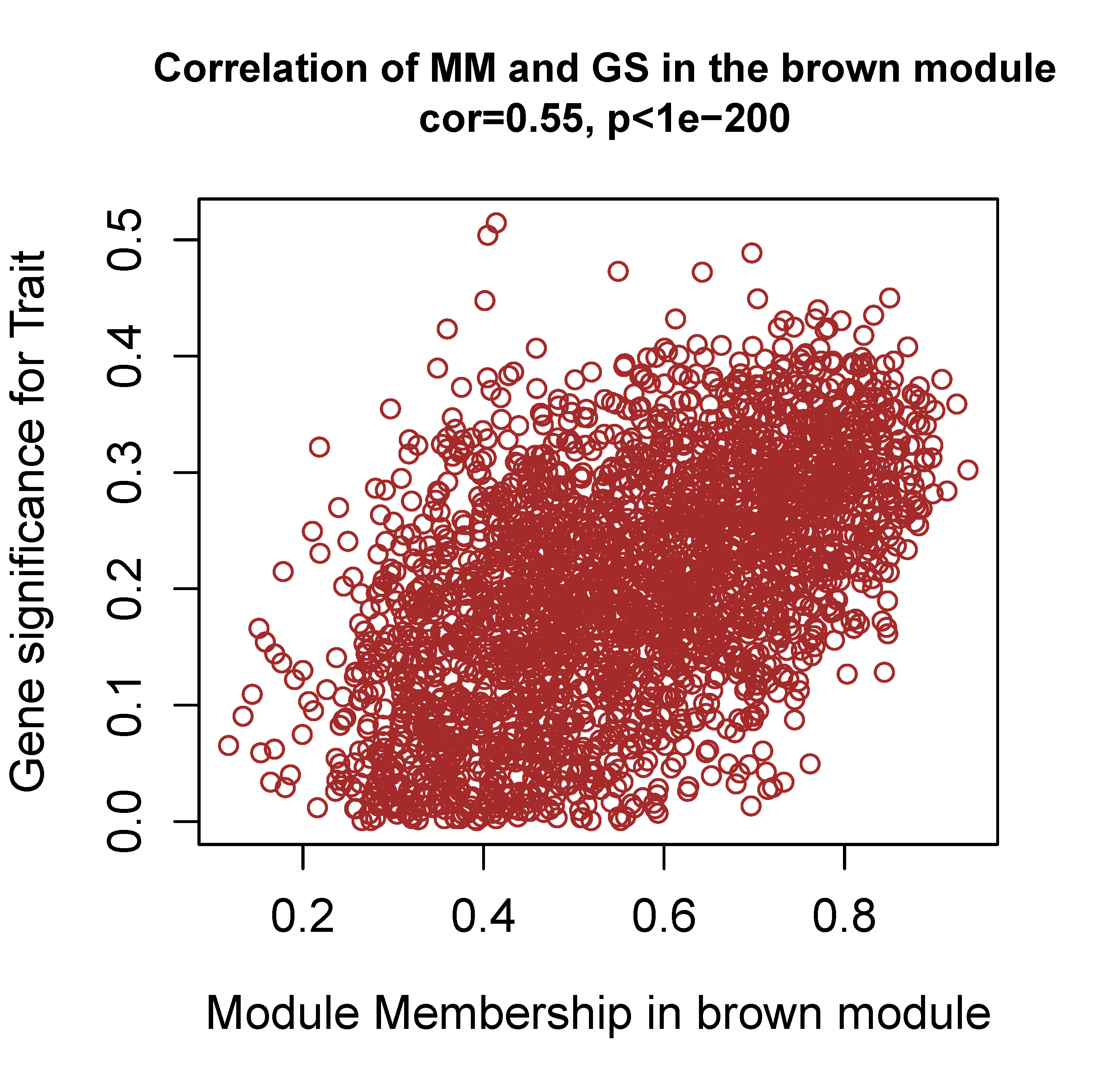

Supplement: Supplementary file 8 [file Image6.tiff]

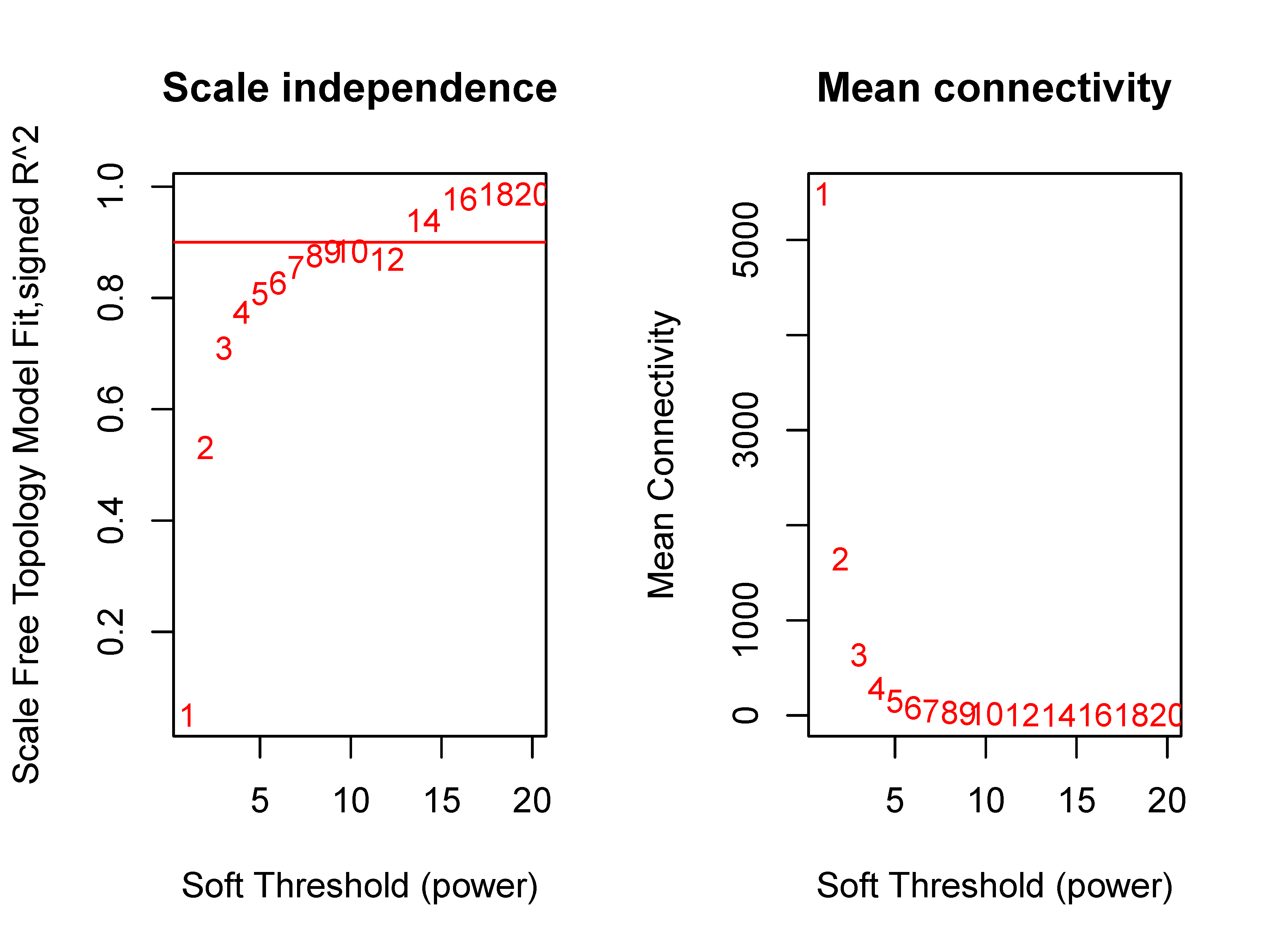

Supplement: Supplementary file 9 [file Image2.tiff]

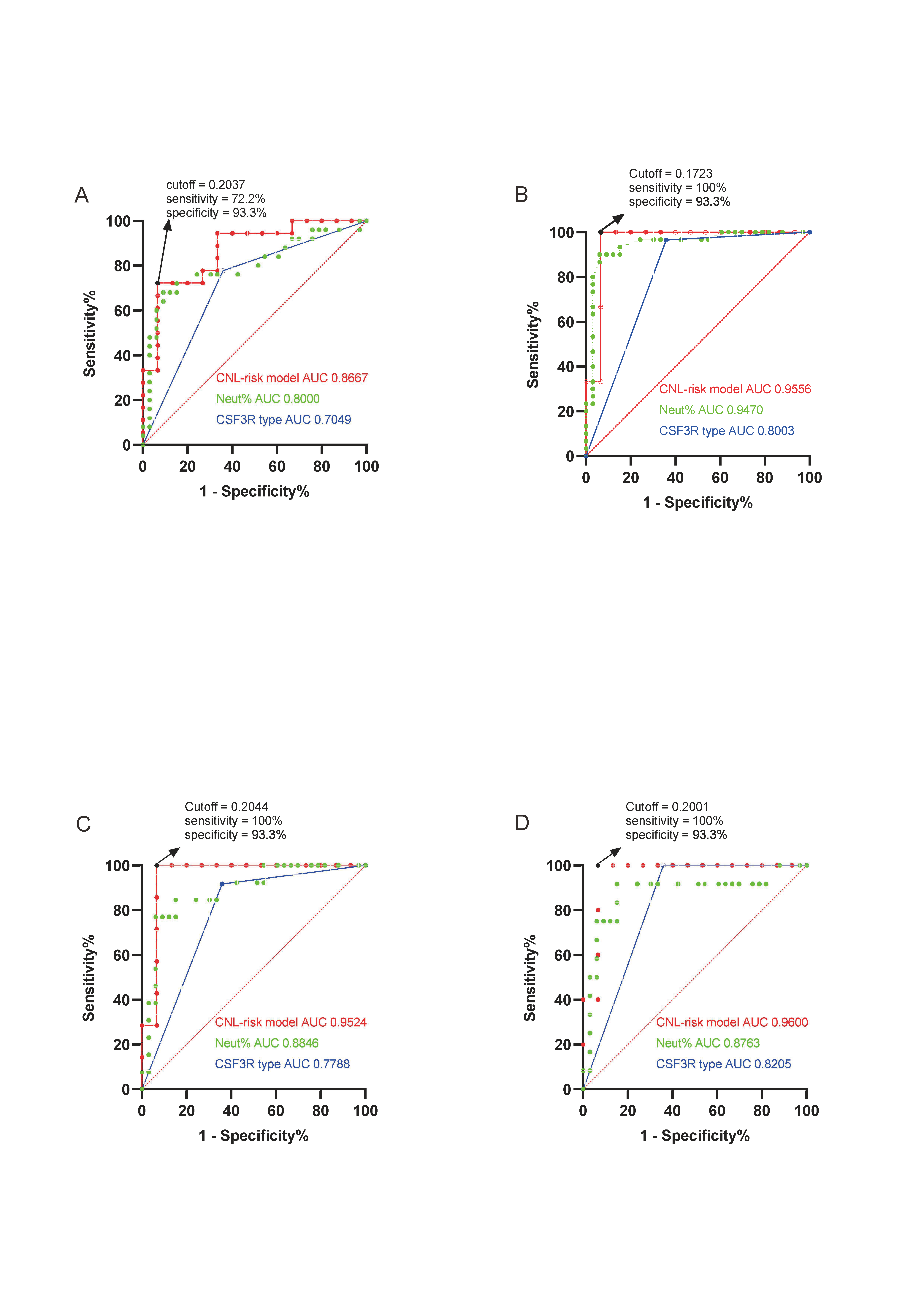

Supplement: Supplementary file 11 [file Image7.tiff]
